# C_60_ Fullerene as Promising Therapeutic Agent for the Prevention and Correction of Skeletal Muscle Functioning at Ischemic Injury

**DOI:** 10.1186/s11671-017-1876-4

**Published:** 2017-02-14

**Authors:** D. M. Nozdrenko, D. O. Zavodovskyi, T. Yu. Matvienko, S. Yu. Zay, K. I. Bogutska, Yu. I. Prylutskyy, U. Ritter, P. Scharff

**Affiliations:** 1 ESC “Institute of Biology and Medicine”, Taras Shevchenko National University of Kyiv, 64 Volodymyrska St., Kyiv, 01601 Ukraine; 2 Lesya Ukrainka Eastern European National University, 13 Volya Av., 43025 Lutsk, Ukraine; 3 Institute of Chemistry and Biotechnology, Technical University of Ilmenau, 25 Weimarer St., 98693 Ilmenau, Germany

**Keywords:** C_60_ fullerene, Skeletal muscle, Ischemia, Muscle contraction dynamics, Biochemical analysis

## Abstract

The therapeutic effect of pristine C_60_ fullerene aqueous colloid solution (C_60_FAS) on the functioning of the rat *soleus muscle* at ischemic injury depending on the time of the general pathogenesis of muscular system and method of administration C_60_FAS in vivo was investigated. It was found that intravenous administration of C_60_FAS is the optimal for correction of speed macroparameters of contraction for ischemic muscle damage. At the same time, intramuscular administration of C_60_FAS shows pronounced protective effect in movements associated with the generation of maximum force responses or prolonged contractions, which increase the muscle fatigue level. Analysis of content concentration of creatine phosphokinase and lactate dehydrogenase enzymes in the blood of experimental animals indicates directly that C_60_FAS may be a promising therapeutic agent for the prevention and correction of ischemic-damaged skeletal muscle function.

## Background

Among muscle pathologies that develop in the skeletal muscles, the ischemic injuries are more than 35% of all injuries of musculoskeletal system [1]. Ischemic-reperfusion injury of skeletal muscle is a major cause of postoperative pathologic complications [2], particularly, the reason of amputations and mortality is acute arterial occlusion [3]. Due to the delivery reduction of oxygen in blood flow through the blood vessels, the nutrients and regulatory substances cannot reach; thus, the muscle decreases. This can lead to a progressive disorder in its metabolic, morphological, and physiological processes.

The main aim in the treatment of the ischemic muscles is the fast recovery of blood flow (reperfusion) in the damaged areas. However, this therapy often leads to new pathophysiological process; reperfusion injury, which also can cause significant damage in the muscle tissue.

At ischemic injury of the skeletal muscle, there is a high correlation between the duration of ischemia and survival of muscle fiber [4]. Despite the fact that different types of fibers in the skeletal muscle differ from the metabolic and functional properties, it has no significant impact on their tolerance to ischemia-reperfusion injuries [5].

At the biochemical level, the ischemic damage of the muscle tissue is a sequence of biochemical reactions, which are initiated by hypoxia after a few minutes of ischemia and occur independently of etiological features due to insufficient blood supply to the muscle [1]. The death of the majority of the muscle cells is a result of chemical substance activation, which are produced during and after ischemia and can be formed within a few days even after the restoration of normal blood flow to the muscles.

It is known that after 2 h of skeletal muscle ischemia and further reperfusion, the concentration of ATP significantly reduced simultaneously with a significant increase in the number of lactate from 25 to 114 mmol/kg of dry weight. And after 3 h of ischemia, the intramuscular supply of ATP is about 5% from baseline, and glycogen pool is depleted by 88% [6]. From a functional point of view, these data indicate that a large number of high-energy phosphate compounds in ischemic-damaged muscle cells are spent to maintain homeostasis (especially during the first hour of ischemia) and, consequently, metabolic causes a significant increase in the fatigue ischemic muscle [7].

It is known that free radicals are a major pathogenic factor in the development of ischemic damage in the muscle tissue [8]. Preliminary biological studies of water-soluble pristine C_60_ fullerenes [9–13] have shown that at low (physiological) concentrations, they do not exhibit acute toxic effects on the normal cells [14–16], they are not allergenic and immunogenic and they able to regulate free-radical processes in the cells and tissues, in particular, neutralize excess free radicals [17, 18]. Consequently, the use of biocompatible and bioavailable C_60_ fullerenes as powerful antioxidants [19] opening up new potential opportunities for the prevention and correction of ischemic-reperfusion pathological processes in the muscle tissue.

The purpose of this study was to assess the impact of water-soluble pristine C_60_ fullerenes on mechanical and kinetic peculiarities of rat skeletal muscle function at ischemic injury, namely: (1) to conduct a quantitative analysis of the activity of ischemic-damaged muscle structures and establish a link between the change in mechano-kinetics of physiological contractions and the level of C_60_ fullerenes action that is necessary for this change and (2) to evaluate the therapeutic effect of C_60_ fullerenes on the time of development of general pathogenesis of muscular system depending on the method of administration (intravenous and intramuscular) in vivo.

## Methods

A highly stable reproducible pristine C_60_ fullerene aqueous colloid solution (C_60_FAS) in concentration 0.15 mg/ml was prepared and characterized according to the protocol [20, 21].

The study was conducted on white male rats of the “Wistar” line weighing 170 ± 5 g. The animals were kept under standard conditions in the vivarium of the ESC “Institute of Biology and Medicine”, Taras Shevchenko National University of Kyiv. Animals had free access to food and water. All experiments were conducted in accordance with the international principles of the European Convention for protection of vertebrate animals under a control of the Bio-Ethics Committee of the abovementioned institution.

All experimental animals were divided into four groups: intact group (animals with saline injection; *n* = 10), control group (animals after ischemia without C_60_FAS injection; *n* = 10), and two experimental groups (animals after ischemia with C_60_FAS injection intravenously (*n* = 10) and intramuscularly (n = 10) immediately after reperfusion). For therapeutic purposes we used C_60_FAS in a concentration of 1 mg/kg because this dose was the most effective at muscular therapy [22, 23].

Anesthesia of animals was performed by intraperitoneal administration of nembutal (40 mg/kg). For muscle ischemia, the branch of the femoral artery of the animal, which provides blood supply of the experimental muscle, was dragged by ligatures. Standard preparation of the experiment also included the cannulation (a. carotis communis sinistra) for the therapeutic administration of C_60_FAS and pressure measurement, tracheotomy, and laminectomy at lumbar spinal cord level. *Soleus muscle* of rat was released from the surrounding tissues and its tendon was cut across in distal part. The ventral roots were cut in places of their exit from the spinal cord for the modulated stimulation of efferents in L7-S1 segments.

The change in muscle contraction force was measured using the original strain gages [22, 23]. To generate stimulus signals, the programmable generator of signals of special form was used.

The study of dynamic properties of muscle contraction was performed under conditions of muscle activation using the modulated stimulation of efferents. Five filaments of ventral roots were cut and fixed on stimulating electrodes, and a special device was used for cyclic sequence distribution of electrical signals via the filaments [22, 23]. The distributed stimulation was allowed to get the monotonous and uniform muscle contraction at low stimulation frequency of individual filaments (50 Hz). Stimulation of efferents in L7-S1 segments was performed by electric impulses of 2 ms, formed by using a pulse generator controlled by ACC through the platinum electrodes. The parameters of stimulated signal were programmed and transmitted from the ACC-CAC complex to generator. A control of external load on the muscle was carried out with the help of original mechanical stimulator [23, 24]. The electromagnetic linear motor was used for perturbation load.

The muscle contraction force was measured at 1, 2, 3, 4, and 5 experimental hours and at 1, 2, 3, 4, and 5 experimental days after ischemia. All received force curves reflect the change in the percentage of control values of the intact muscles, which were taken as 100%.

Level of content of creatine phosphokinase (CPK) and lactate dehydrogenase (LDH) enzymes in the blood of experimental animals, as the markers of ischemic injury of the skeletal muscle, was determined by using a clinical equipment.

The experimental data were stored and analyzed by statistical processing of the results using standard software packages Excel and Origin 8.0. All results were expressed as mean ± SEM. The significance of differences of baseline values between control and experimental groups was evaluated by *t* test. A value of *p* < 0.05 was considered statistically significant.

## Results and Discussion

### Biomechanical Study

Kinetics of muscle fatigue and change of the force response in each flow of muscle contraction induced by stimulation successive pools are important characteristics of the pathogenesis of muscle ischemic injury study. Under normal conditions, the fatigue changes during the contraction of the *soleus muscle* detected only after 5–6 h stimulation [25].

The main processes, which initiate a cascade of ischemic pathologies in the damaged muscle, occur in the first hours after reperfusion [26]. Based on this, the first step in research is to examine the change in the dynamics of the contractile process in the first 5 h after reperfusion of the ischemic *soleus muscle*. Comparing intravenous and intramuscular administration of C_60_FAS, we tried to determine the optimal method of its administration to achieve maximum therapeutic effect.

**Fig. 1:**
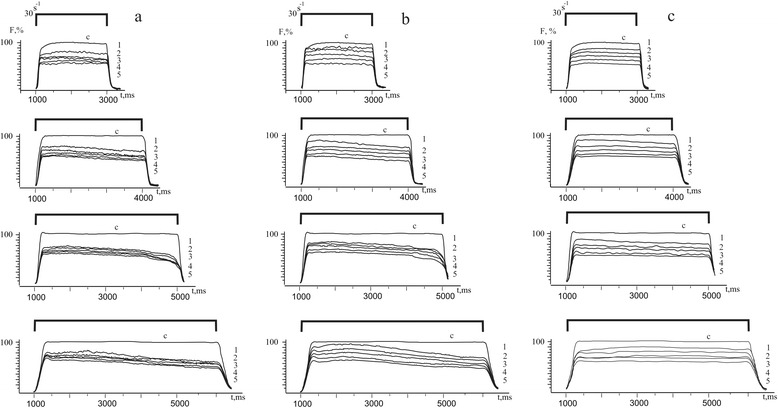
Force generation curves (F, %) of the ischemic *soleus muscle*: **a** control (without affecting C_60_FAS; *n* = 10); **b** intravenous injection of C_60_FAS (dose 1 mg/kg; *n* = 10); **c** intramuscular injection of C_60_FAS (dose 1 mg/kg; *n* = 10). *1*, *2*, *3*, *4*, *5* hours after reperfusion of the muscle

Figure 1 shows change in the *soleus muscle* force response during the first 5 h after its reperfusion under activating stimulus pools with duration from 2 to 5 s.

**Fig. 2:**
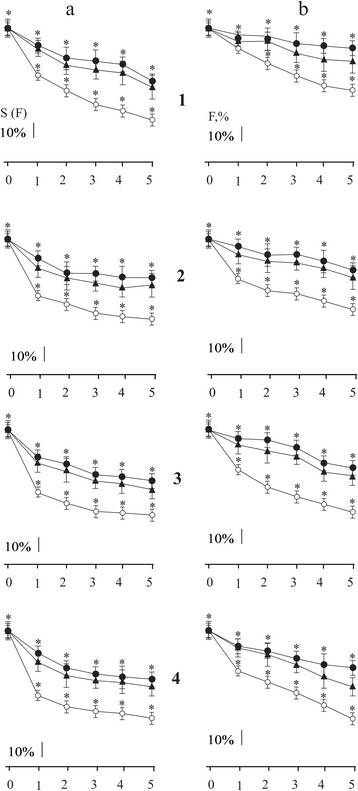
Change of the integrated power S(F) (**a**) and maximal force of contraction (F) (**b**) (as a percentage of control values adopted by 100%) of the ischemic *soleus muscle* at different duration of modulated stimulation: *1*, *2*, *3*, *4* stimulation *2*, *3*, *4*, *5* s, respectively; (○) control (without affecting C_60_FAS; *n* = 10); (▲) intravenous administration of C_60_FAS (dose 1 mg/kg; *n* = 10); (●) intramuscular administration of C_60_FAS (dose 1 mg/kg; *n* = 10). *0*, *1*, *2*, *3*, *4*, *5* hours after reperfusion muscle. *p < 0.05

In the control (without affecting C_60_FAS), reduction of maximal force responses not only with an increasing time after ischemia but also with increasing duration of irritating stimulus signal is observed (Fig. 2). In case of therapeutic administration of C_60_FAS, the reduction of force response with increasing of time irritating signal is negligible and depends mainly on time after reperfusion (Fig. 2). It is important to note that in this case the method of administration of C_60_FAS had no significant value.

The registration of such important biomechanical parameter as integrated power (it’s calculated by total area, which describes the force curve) found the similar results (Fig. 2): the integrated power reduction as with an increasing time after reperfusion and with increasing duration stimulation signal is largely compensated by the influence of C_60_FAS regardless of its method of administration. It is also important to note that the protective effect of C_60_FAS manifests on the first hours of ischemic muscle injury, during which the initiation of the main stages of ischemic destruction of the muscle tissue takes place.

**Fig. 3:**
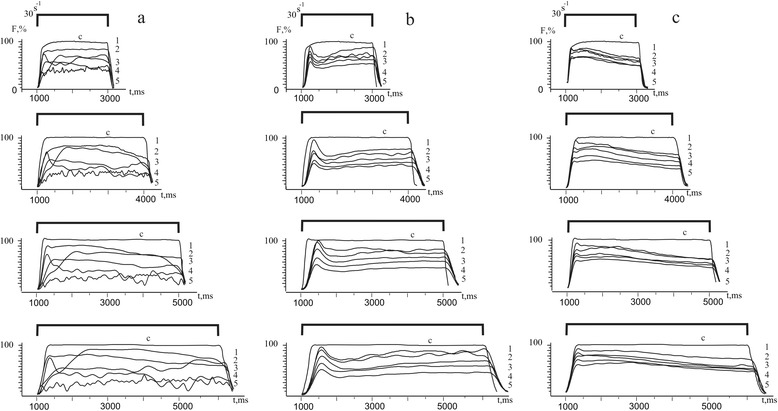
Force generation curves (F, %) of the ischemic *soleus muscle*: **a** control (without affecting C_60_FAS; *n* = 10); **b** intravenous injection of C_60_FAS (dose 1 mg/kg; *n* = 10); **c** intramuscular injection of C_60_FAS (dose 1 mg/kg; *n* = 10). *1*, *2*, *3*, *4*, *5* days after reperfusion of the muscle

**Fig. 4:**
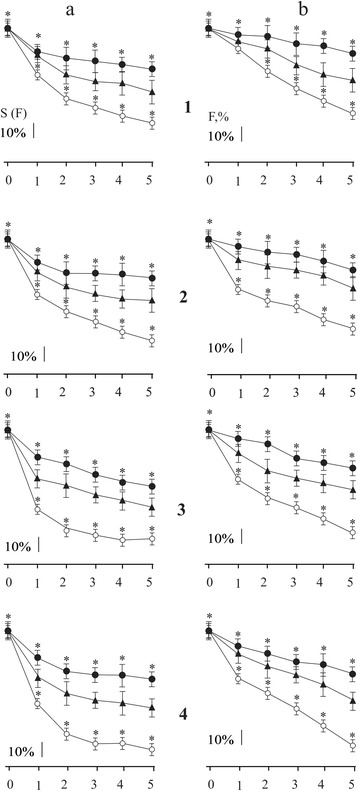
Change of the integrated power S(F) (**a**) and maximal force of contraction (F) (**b**) (as a percentage of control values adopted by 100%) of the ischemic *soleus muscle* at different duration of modulated stimulation: 1, *2*, *3*, *4* stimulation *2*, *3*, *4*, *5* s, respectively; (○) control (without affecting C_60_FAS; *n* = 10); (▲) intravenous administration of C_60_FAS (dose 1 mg/kg; *n* = 10); (●) intramuscular administration of C_60_FAS (dose 1 mg/kg; *n* = 10). *0*, *1*, *2*, *3*, *4*, 5 days after reperfusion of the muscle. *p < 0.05

Based on the fact that muscle contraction is a dynamic vibrational process of mutual reactions, one can assume that in the conditions of pathological changes in muscle fibers caused by ischemia, there should be optimum stimulation parameter ratio, which can involve the maximum number of sarcomere structures for the optimal muscle contraction. Although the heterogeneous composition of skeletal muscle contractile apparatus is difficult to assess the damage of each individual component, the overall picture of the pathological process can be traced by measuring the level of changes of maximum force contraction for several days (Figs. 3 and 4). In control, the muscle activity had a tendency to linear force reduction response with an increasing time after reperfusion that may indicate the development of muscle fatigue. But unlike the fatigue process starting from the 2nd day of the experiment, the force curves contain the pronounced fluctuation components. If the power drop is caused by reduction of molecular generators of power, i.e., by reduction of working cross-bridges; then, in the case of fluctuation contractions, the damages should take place in almost all contractile components of the muscle cells. So, in this case, one can speak only about relatively similarity of force responses during fatigue and induced ischemia just in the early stages of pathological process. The significant dependence of dynamic characteristics of contraction on the activity of the main types of proprioceptors significantly complicates the control of the motor activity of the damaged muscle from the central nervous system (CNS) in case of uncontrolled fluctuation responses of ischemic-damaged muscle even on a simple stimulus signal. Elimination of these vibration components of muscle contraction with action of C_60_FAS (regardless of the therapeutic administration method) is a very important feature of its protective effect (Fig. 3).

With using modulated stimulation, the quantitative and qualitative differences in the contraction of ischemic rat’s *soleus muscle* in control and with C_60_FAS were observed (Fig. 4). In control, the value of the maximum force and integrated power of muscle contraction decreased with an increasing time after reperfusion as well as the duration of stimulation (Fig. 4). Therapeutic administration of C_60_FAS found the significant protective effect on contraction force characteristics that were studied as follows: the most pronounced protective effect was observed on the 5th day after ischemia and at maximum 5 s of stimulation; C_60_FAS protective effect on the maximum force response was 30–35%, and the integrated power—over 50% compared to control. In this case, the difference between protective effects of C_60_FAS depending on the method of administration was observed. Thus, intramuscular injection of C_60_FAS showed 10–15% more protective effect on muscle force response in comparing with the intravenous administration of C_60_FAS.

Differences in increasing force and integrated power of ischemic-injured muscles during intravenous and intramuscular administration of C_60_FAS (Figs. 1, 2, 3, and 4) indicate the complexity of the molecular mechanisms of muscle contraction, which are probably different in implementing antioxidant properties of C_60_ fullerenes. Obviously, at the therapeutic injection of C_60_FAS directly into the damaged muscle, the concentration of C_60_ fullerenes are much higher than in the area of inflammation compared with intravenous administration of C_60_FAS. Thus, it can be argued on the realization of concentration dependence of the protective effect of C_60_ fullerenes on the maximum force contraction and integrated power of the ischemic injured muscles.

Observed high correlation between the duration of ischemia and muscle fiber survival [4] can be one of the main factors reducing the maximum force response with time increasing after ischemia not only due to the decrease of muscle fibers survival but also due to the increased rigidity of the muscle (due to an increase of its collagen structures). After 3 h of ischemia, the muscle necrotic changes and nervous degradation occur. The amount of muscle tissue necrosis may be up to 60% [27]. In this case, the therapeutic action of C_60_FAS will not have a positive effect. Thus, C_60_FAS, use as a therapeutic agent for ischemic muscle damage, will have a pronounced beneficial effect mainly on the early stages of this disease.

According to modern theories of motor control in the development of muscle pathologies, CNS organizes the limb movements so as to reduce the number of degrees of freedom, which correspond to movements of the individual segments. The reason for this decrease is the synergies (involving functional activity of intact or partially damaged muscle fibers), which leads to complications of central management program movements, thus compromising control over the implementation of purposeful movements [28]. Because the structure of the dynamic component of stimulation (ratio of its amplitude to duration) determines the speed and range of motion, the changing nature of efferent activity realization of ischemic muscle results in errors in the positioning accuracy of the joint. By performing even simple movements, there is a possibility to establish causal links between the mechanical activity of ischemic-injured muscles of the joint and key dynamic parameters of movement. The accuracy of this analysis rises via detailed study of before tetanic areas of muscle contraction with simultaneous control of mechanical movement parameters [29]. Therefore, the studying changes in the dynamics of ischemic-damaged muscle contraction on before tetanic areas allow to detect the level of muscle damage and effectiveness of therapeutic action of C_60_FAS.

**Fig. 5:**
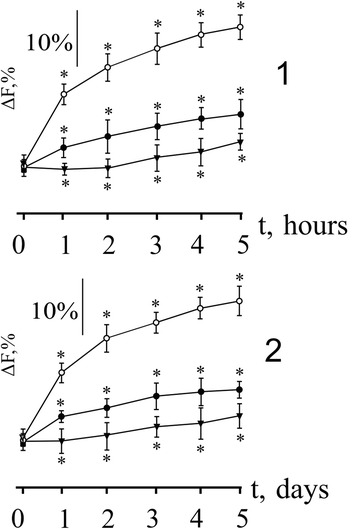
Change of speed attaining maximum force response (ΔF, %) of the ischemic *soleus muscle*: (○) control (without affecting C_60_FAS; *n* = 10), (▲) intravenous injection of C_60_FAS (dose 1 mg/kg; *n* = 10), (●) intramuscular injection of C_60_FAS (dose 1 mg/kg; *n* = 10). *1* and *2* hours and days, respectively, after reperfusion of the muscle. *p < 0.05

Figure 5 shows changes in achieving speed of maximum force response of the ischemic muscle in dependence of time after ischemia: in the first 5 h and next 5 days after reperfusion. In control, after 1 h reperfusion, the reduction of maximum force and an increase of time to achieve it are observed. The therapeutic application of C_60_FAS essentially adjusts the dynamics of the force curves: a clear separation of dynamic and stationary parts of the contraction occurs. It should be noted that this effect is independent of the manner of C_60_FAS administration. A more pronounced effect of C_60_FAS on before tetanic area muscle contraction and less on the maximum force response (Fig. 5), in our opinion, is connected with the beginning of irreversible pathological changes in the generation of force contraction on the most vulnerable before tetanic areas in the early stages of ischemic lesion of muscle cells. This is due to the uneven destruction of different molecular components of the contractile apparatus of muscle, which are activated in different phases of contraction and therefore face different impact of C_60_FAS. However, even a slight protective effect of C_60_FAS on tetanic areas of muscle contraction is essential in the first hours after launch of ischemic cascade of pathological processes and C_60_ fullerenes have to slow down their, particularly, neutralizing free radicals in early ischemic injury muscle.

Research of speed change achieving maximum level of force within 5 days in control (Fig. 5) found a direct relationship between the speed reduction and time after reperfusion. Therapeutic administration of C_60_FAS significantly corrected this parameter: its reduction after the 1st day after ischemia was not changed significantly over the next 4 days of experiment. Intravenous administration of C_60_FAS demonstrated better protective effect (on 10%) compared to intramuscular administration of C_60_FAS.

### Biochemical Study

Most characteristic biochemical compounds which, on the one hand, easily identified in clinical conditions and, on the other hand, the content of which changes significantly upward in patients with ischemic injuries, are CPK and LDH [30].

CPK is contained in high concentration in the skeletal muscles, and body consumes it rapidly by increasing physical activity. If the damage of myocytes happens, CPK diffuses from them, thus increasing its activity in the blood. Therefore, determining the activity of CPK in the blood is a sensitive diagnostic test for the manifestation of ischemic damage in the muscle tissue [31].

**Fig. 6:**
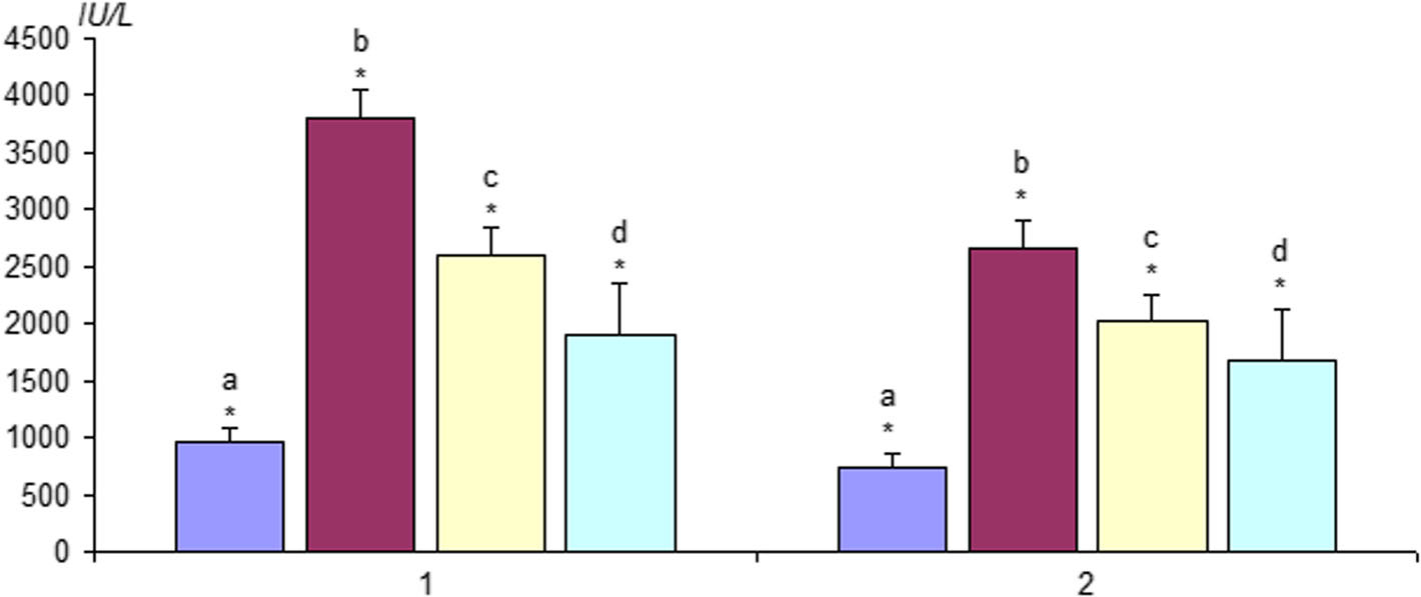
Change in the content of creatine phosphokinase (1) and lactate dehydrogenase (2) in the ischemic *soleus muscle* 5 h after reperfusion: **a** intact animals (*n* = 10), **b** control (without affecting C_60_FAS; *n* = 10); **c** intravenous injection of C_60_FAS (dose 1 mg/kg; *n* = 10); **d** intramuscular injection of C_60_FAS (dose 1 mg/kg; *n* = 10). *p < 0.05

**Fig. 7:**
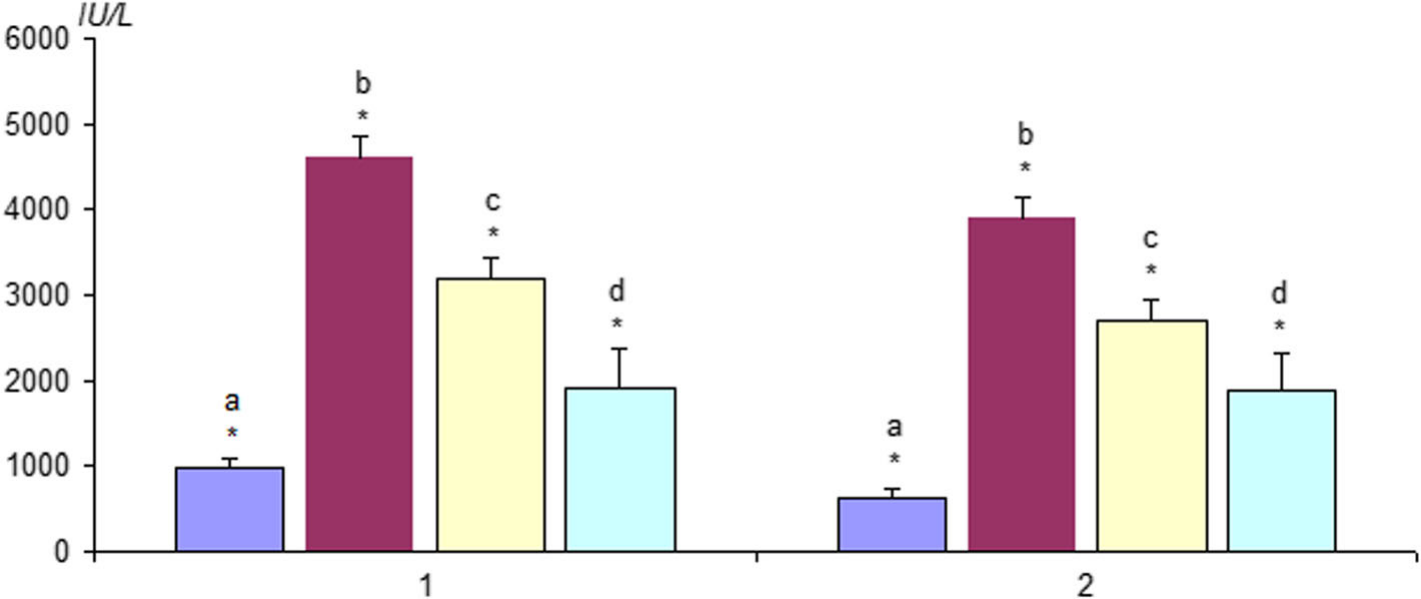
Change in the content of creatine phosphokinase (1) and lactate dehydrogenase (2) in the ischemic *soleus muscle* after 5 days after reperfusion: **a** intact animals (*n* = 10), **b** control (without affecting C_60_FAS; *n* = 10), **c** intravenous injection of C_60_FAS (dose 1 mg/kg; *n* = 10), **d** intramuscular injection of C_60_FAS (dose 1 mg/kg; *n* = 10). *p < 0.05

LDH participates in the processes of oxidation of glucose and the formation of lactic acid. It is contained in almost all organs and tissues of the human, especially a lot of it in the muscles. In the conditions of hypoxia, LDH causes a feeling of muscle fatigue and breaks the process of tissue breathing. Blood tests for CPK and LDH used in the clinic for the rapid identification of diseases associated with ischemic injuries of the muscular system [32]. Determination of the levels of these enzymes in the blood of tested animals showed accurate tendency to their reduction after therapeutic administration of C_60_FAS after the first 5 h (Fig. 6) and 5 days after ischemia (Fig. 7).

Considering that at ischemic injury of the skeletal muscle, the reactive oxygen species (ROS) are of the most destructive danger, the use of C_60_ fullerenes as powerful antioxidants should significantly improve muscle tolerance to ischemia and expedite postoperative recovery [33].

The observed effects above may be related to the fact that 2 h ischemia-reperfusion of the *soleus muscle* significantly reduces the concentration of ATP with significant increase in lactate. It is known that, for a 3 h ischemia, ATP depletion is about 95%, and glycogen depletion is 88% [6, 7]. In addition, a large number of high-energy phosphates are spent by the damaged muscle cell to maintain hemostasis and, as a result, the metabolic disorder leads to greater muscle fatigue. At the same time, literature data indicate that ROS (for example, superoxide anion and hydroxyl radical) are a major pathogenic factor in ischemia-reperfusion tissue damage. ROS initiate the lipid peroxidation, direct inhibition of mitochondrial respiratory chain enzymes, inactivation of glyceraldehyde-3-phosphate dehydrogenase, ATPase inhibition activity, inactivation of membrane sodium channels, etc. It was shown that modified C_60_ fullerenes can be considered as a powerful ROS absorber of ischemia-reperfusion-induced injury of small intestine [34]. Also, the ability of C_60_ fullerene derivatives to reduce the ischemia-reperfusion lung injury was demonstrated [35, 36]. In this regard, the protective effect of C_60_FAS on the fatigue processes of the ischemic-damaged muscle can be directly linked to the strong antioxidant properties of pristine C_60_ fullerenes.

## Conclusions

The results of this study can be united in the following paragraphs:

1. A pronounced protective effect of C_60_FAS on the contractile dynamics of *muscle soleus* ischemic injury was reliably established.
2. It was shown that intravenous and intramuscular injections of C_60_FAS have different therapeutic effects: the intravenous injection of C_60_FAS is optimal for correction of speed macroparameters of contraction at ischemic muscle damage; the intramuscular injection of C_60_FAS demonstrates more pronounced protective effect in movements associated with the generation of maximum force responses or prolonged contractions caused by increasing levels of muscle fatigue. It must be emphasized that protective effect of C_60_FAS is also important to correct the accuracy of the joint position of the injured limb, since for precision positioning and fine motor skills of limbs, extremely important is the ability to hold the tetanic contraction regime by the antagonist muscles, the implementation of which is lost during ischemic pathology.
3. The use of biocompatible water-soluble pristine C_60_ fullerenes considering prominent antioxidant properties and lack of data of acute and chronic toxicity open new possibilities in the therapy and prevention of ischemic pathologies.

## Abbreviations

C_60_FAS: C_60_ fullerene aqueous colloid solution
CNS: Central nervous system
CPK: Creatine phosphokinase
LDH: Lactate dehydrogenase
ROS: Reactive oxygen species
